# Age Impaired endothelium-dependent vasodilation is improved by resveratrol in rat mesenteric arteries

**DOI:** 10.20463/jenb.2016.03.20.1.2

**Published:** 2016-03-31

**Authors:** Semil S Gocmez, Philip J Scarpace, Melissa A Whidden, Benedek Erdos, Nataliya Kirichenko, Yasemin Sakarya, Tijen Utkan, Nihal Tumer

**Affiliations:** 1 Geriatric Research, Education, and Clinical Center, North Florida/ South Georgia Veterans Health System, Gainesville, FL 32608United States; 2 Department of Pharmacology and Therapeutics, College of Medicine, University of Florida, Gainesville, FL 32610United States; 3 Department of Kinesiology, West Chester University, West Chester, PA 19383United States; 4 Department of Pharmacology, University of Vermont, Burlington, VT 05405United States; 5 Department of Pharmacology, Kocaeli University, Kocaeli, 41380Turkey

**Keywords:** Aging, resveratrol, adrenal, catecholamines, mesenteric artery

## Abstract

**[Purpose]:**

To determine whether resveratrol improves the adverse effects age on vascular function in mesenteric arteries (MAs), and diminishes the hyperactivity in adrenal gland with age.

**[Methods]:**

Male F344 x Brown Norway rats were assigned to 6-month control (YC), 6-month resveratrol (YR), 24-month control (OC) and 24-month resveratrol (OR). Resveratrol (15 mg/kg) was provided to resveratrol groups in drinking water for 14 days.

**[Results]:**

Concentration response curves to phenylephrine (PE, 10^-9^-10^-5^M), acetylcholine (Ach, 10^-9^-10^-5^M) and resveratrol (10^-8^-10^-4^M) were evaluated in pressurized isolated MAs. The Ach concentration-response curve was right shifted with maximal response diminished in OC compared with YC rats. These effects were reversed by resveratrol treatment. The resveratrol-mediated relaxant responses were unchanged with age or resveratrol suggesting an endothelium-independent mechanism. Resveratrol tended to increase endothelial nitric oxide synthase; caused no effect on copper-zinc superoxide dismutase; and normalized the age-related elevatation in DβH and NPY levels in adrenal medulla, two indicators of sympathetic activity

**[Conclusion]:**

These data indicate that resveratrol reverses age-related dysfunction in endothelium-dependent vasodilation in MAs and partially reverses hyperactivity of adrenomedullary function with age. This treatment may have a therapeuticpotential in the treatment of cardiovascular diseases or hypertension in the elderly.

## INTRODUCTION

Aging is associated with phenotypic and functional changes in vascular structure and functionand there is an age-related increase in the prevalence of hypertension, all of which elevate the risk for cardiovascular disease^1-3^. There is profound evidence for an increase in intima-media-thickness and vascular stiffness not only during healthy aging but also induced by cardiovascular risk factors^4^. Endothelial dysfunction which characterizes vascular aging is strictly associated with decreased nitric oxide (NO) bioavailibility, resulting in impaired vasodilatation, increased plaque formation and thrombosis ^5-7^. Several mechanisms are involved in reduced NO availability in aging. First, endothelial nitric oxide synthase (eNOS) activity and therefore eNOS derived NO production decline with increasing age ^8^; second, excess reactive oxygen species (ROS) produced by arteries during aging combine with NO to form peroxynitrite, a powerful oxidant, which is increased in the arterial media of aging vessels ^9-11^.

With aging, there is dysregulation of blood pressure, and the age-related increases in hypertension are associated with an increase in sympathetic nervous activity^12-14^, and sustained elevation of catecholamine biosynthesizing enzymes in the adrenal medulla and peripheral sympathetic ganglia. Protein levels and enzyme activity of tyrosine hydroxylase (TH), the rate-limiting enzyme in catecholamine biosynthesis, are elevated in the adrenal medulla of senescent rats compared with younger animals^15-18^. In addition, protein levels of another key catecholamine synthesizing enzyme, dopamine beta-hydroxylase (DβH) as well as NPY, a peptide, co-localized and co-released with catecholamines in central and peripheral nerves as well as the adrenal medulla, are also upregulated in the adrenal medulla with age^19-22^. Adrenal medullary levels of TH, DβH, and NPY are key indicators of sympathetic nervous system activity.

Resveratrol (3,5,4-trihydroxy-trans-stilbene) is a polyphenol phytoalexin, found in grape skins and red wine^23,24^. Accumulating reports have shown that resveratrol can prevent or slow the progression of a variety of diseases, including cancer, ischemic injuries, Alzheimer’s, as well as cardiovascular disease^25,26^. The mechanism of cardiovascular benefits probably include vasorelaxant, antioxidant, and antiplatelet effects of resveratrol^2^7. In vitro studies have demonstrated that resveratrol has vasodilatory effects when applied to different isolated artery segments at pharmacological concentration. In organ chambers in vitro, resveratrol inhibits the contractile response to noradrenaline and causes relaxation of the phenylephrine-precontracted rat aorta^28^. The vasorelaxant activity of resveratrol has also been observed in isolated mesenteric and uterine arteries of guinea pigs^29^, in mesenteric resistance arteries of lean and obese rats^30^, in porcine coronary arteries^31^ and human internal mammary arteries^32^. Furthermore, resveratrol improves endothelial function *in vivo*. Endothelial dysfunction is an early event in the development of atherosclerosis and is present even before structural changes occur in the vasculature. All major risk factors for atherosclerosis such as hyperlipidemia, diabetes, hypertension, increasing age, and smoking are associated with endothelial dysfunction^7,33^. It was shown that oral treatment with resveratrol resulted in improvement in endothelial function in hypertensive rats^34^, diabetic rats and mice^35^, hypercholesterolemic rabbits^36^ as well as healthy rats^37,38^. These studies indicate the potential of resveratrol for health promotion and disease prevention. Therefore, in this study we aimed to evaluate whether resveratrol reverses the adverse effects of age on vascular function in mesenteric arteries (MAs) and adrenal medularry indicators of sympathetic activity. To this end, in young and old rats, we determined the vasorelaxant effects of resveratrol in isolated mesenteric arteries, eNOS and CuZnSOD in aorta, and the effects of resveratrol on TH, DβH, and NPY levels in adrenal medulla.

## METHODS

### Animal Preparation

Six-and 24-month-old male Fischer 344xBrown Norway rats were obtained from Harlan (Indianapolis, IN). Upon arrival, rats were examined and remained in quarantine for one week. Animals were cared for in accordance with the principles of the Guide to the Care and Use of Experimental Animals, and all procedures were approved by the local Institutional Animal Care and Use Committee (ACORP # 9811-010/Protocol # 003, dated 16 December 2008). Rats were housed individually with a 12:12 h light:dark cycle (06:00 to 18:00 hr) with water available *ad libitum*. The rats were randomly divided into 4 groups of 8 animals in each group; young control (YC), young resveratrol (YR), old control (OC) and old resveratrol (OR) rats. Resveratrol (90 mg/L, approximately 15 mg/kg) was given to YR and OR groups in their drinking water (bottles were covered and protected from light) for 14 days *ad libitum*. The concentration of resveratrol was chosen on the basis of previous *in vivo* observations in rats, rabbits, and mice in which the biological effects of resveratrol were observed at concentrations ranging from 3-22 mg/kg administered orally^25,35,36^. Resveratrol was obtained from Sigma (St. Louis, MO, USA) and prepared fresh every day during the experiment. Resveratrol was dissolved in absolute ethanol and diluted with water to a concentraion of 90mg/L. The fresh drinking water (with or without resveratrol) was provided once a day and total water intake was recorded daily. Body weights of the animals were recorded at the beginning and ending of the resveratrol treatment. Experiments were carried out on day 15 following resveratrol or vehicle consumption.

### Tissue Harvesting and Preparation

After 2 weeks, rats were over-anesthetized with pentobarbital (120 mg/kg i.p.) and blood was collected through cardiac puncture. The mesenteric artery bed was removed immediately and placed in cold, oxygenated modified Krebs-Ringer bicarbonate solution (in mM: 119 NaCl, 4.7 KCl, 24 NaHCO3, 1.18 KH2PO4, 1.17 MgSO4, 0.026 EDTA, 1.6 CaCl2, and 5.5 glucose). Next, the circulatory system was perfused with 60 ml of cold saline. The aorta and adrenal medula were rapidly dissected, immediately frozen in liquid nitrogen and stored at -80°C until subsequent analysis.

### Isolation and Cannulation of MAs

These methods have been described previously in detail^39^. Briefly, with the aid of a dissecting microscope, a section of the MA (2 mm in length) was transferred to a vessel chamber and mounted and secured between two glass micropipettes with a 10-0 ophthalmic suture. The vessel chamber was transferred to an inverted light microscope stage coupled to a video dimension analyzer and a strip-chart recorder. The video dimension analyzer was connected to both a video monitor (for visualization of the vessel) and a strip-chart recorder for constant recording of the intraluminal diameter of the vessel. Oxygenated 20% O_2_-5% CO_2_-75% N2Krebs’ solution maintained at 37°C, was continuously circulated through the vessel bath. In addition, the lumen of the vessel was filled with Krebs solution through the micropipettes and maintained at a constant pressure of 80 mmHg. To facilitate the analysis of dilatory responses, appropriate amounts (10^-9^- 10^-5^M) of phenylephrine (PE) were added to constrict the arteries to about 40% of their initial diameter. This provided a wider range for analysis of diameter changes during relaxation and provided a similar level of initial vascular constriction. Experimental protocols were not initiated

### Measurement of vascular responses

Concentration-response curves to PE (10^-9^-10^-5^M) or to acethylcholine (Ach 10^-9^-10^-5^M) and resveratrol (10^-8^-10^-4^M) in vessels preconstricted with PE were evaluated in pressurized isolated mesenteric arteries. The relaxant responses to sodium nitroprusside (SNP) (10^-5^M) were obtained after Ach responses. Relaxant responses were expressed as percentage of the precontraction response to PE.

### Western Blot analysis

The aortas were homogenized to examine copper-zinc superoxide dismutase (CuZnSOD) and eNOS, whereas adrenal medullae samples were used for the assesment of tyrosine hydroxlase (TH) and dopamine beta-hydroxylase (DβH) using Western blot analysis. Tissue samples were homogenized in 50 mM Tris (pH 7.0) with leupeptin and protein content was assessed by the DC protein assay (Bio-Rad, Hercules, CA). An equal amount of protein for each sample was separated by polyacrylamide gel electrophoresis via 12.5% gradient polyacrylamide gels containing 0.1% sodium dodecyl sulfate for 1 h at 100 mA. After electrophoresis, the proteins were transferred to nitrocellulose membranes at 90 V for 1.5h. To control for protein loading and transfer differences, membranes were stained with Ponceau S and analyzed. The membranes were washed and subsequently blocked with 5% skimmed milk in Tris-buffered saline containing 0.1% Tween 20 for 1 h at room temperature and subsequently incubated overnight at 4°C with a primary antibody. This step was followed by incubation at room temperature with a secondary antibody directed against the primary for 1 h. All bound antibodies were detected by chemifluorescence (ECL Plus Western Blotting Detection System; GE Healthcare), scanned (Storm 860 Phosphorimager Scanner; GE Healthcare) and analyzed using Image Quant software.

### Primary antibodies

Copper zinc superoxide dismutase (Anti-CuZnSOD, Calbiochem/ EMD Chemicals Inc. Gibbstown, NJ USA. Catalog number 574597); (Anti-eNOS, AbCam Cambridge, MA USA. Catalog number ab5589); TH (Pel Freez Biologicals, Rogers, AR); and DβH (Novus Biologicals, Littleton, CO).

### Reverse transcriptase-PCR for NPY in adrenal medulla

NPY mRNA expression was identified in the adrenal medulla by using relative quantitative reverse transcriptase-PCR through the use of Quantum RNA 18s Internal Standards kit (Ambion, Austin, TX). PCR was performed by multiplexing NPY primers (sense 5’-ATGGGGCTGTGTGGACTGACC; antisense, 5’-GTCAGGAGAGCAAGTTTCATTT) and 18S primers. The optimum ratio of 18S primer to competimer was 1:9. PCR was performed at 94°C denaturation for 60 s, 59°C annealing temperature for 60 s and 72°C elongation temperature for 60 s for 23 cycles.

### Statistical Analysis

All data are expressed as mean ± SE. Amplitude of vascular relaxations were calculated as a percentage of preconstriction values. Concentration-response curves were evaluated at each concentration for differences between treated and untreated arteries from young and old animal groups. Differences between experimental groups were evaluated using of two-way analysis of variance, followed by Bonferroni’s post hoc test. The criterion for significance was p<0.05.

## RESULTS

### Animal body weight and resveratrol intake

Mean body weight was significantly higher in the old rats, and the two-week treatment with resveratrol did not alter mean body weight in either age group (Table 1). Resveratrol was administered in the drinking water. Water consumption was greater in the aged compared with the young rats, and thus, the daily dose of resveratrol consumed by the old rats was 33% greater than that consumed by the young. However daily dose of resveratrol adjusted for body weight was nearly identical in the young and old (Table 1).

**Table 1.** Body weight, water consumption and resveratrol consumption.

	Body Weight (initial,g)	Body Weight (final, g)	Water Intake (ml/day)	Resveratrol Intake (mg/day)	Resveratrol (mg/day/kg)
Young Control	356 ± 3.2	362 ± 3.1		0	0
Young Res	359 ± 9.4	362 ± 3.9	60.3 ± 2.6	5.42 ± 0.23	15.0 ± 0
Old Control	545 ± 1.6a	546 ± 1.3		0	0
Old Res	554 ± 1.0a	552 ± 2.0	89.8 ± 2.8*	8.08 ± 0.25*	14.6 ± 0

Data represent the mean ± SE of 8 rats per group. Resveratrol intake calculated from water consumption times resveratrol concentration in water (90mg/L).

*P<0.0001 for difference with age by t-test.

### Vascular reactivity

**Figure 1.**
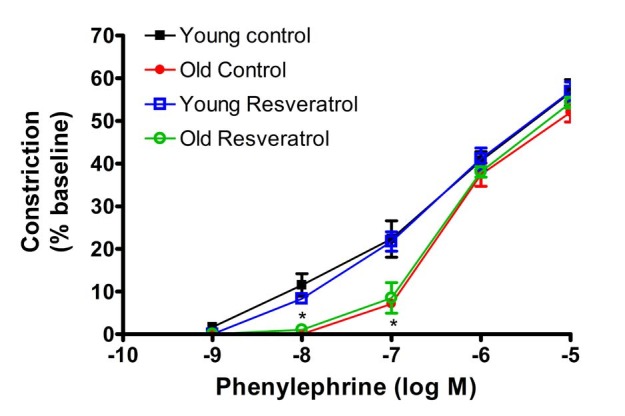
Cumulative dose-response curves to phenylephrine-dependent constriction in mesenteric arteries

PE contracted the cannulated MAs in a concentration-dependent manner with decreased sensitivity demonstrated in the aged rats. Constriction at 10^-8^M and 10^-7^M PE were significantly diminished in aged rats with the receptor activation constant Kact of 2x10^-7^M and 5x10^-7^M, respectively for young and old (Figure 1). There was no change in sensitivity with resveratrol treatment, and maximal constriction with PE was similar in all four groups. Young MAs constricted to 40.86 ± 2.56% and 39.13 ± 3.29% of the initial diameter in the control and resveratrol groups, respectively, while old control MAs constricted to 38.50 ± 2.67% and old resveratrol MAs to 38.33 ± 1.33%.

**Figure 2.**
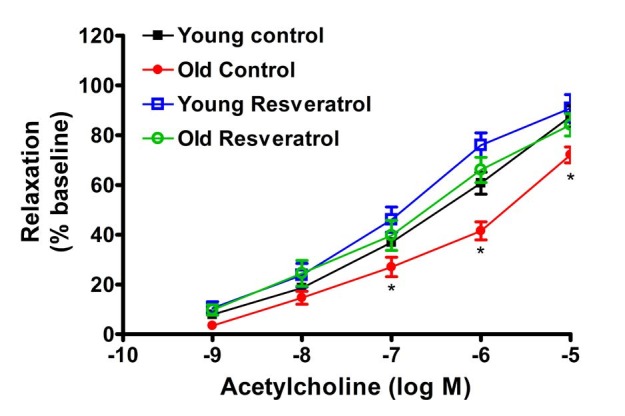
Cumulative dose-response curves to acetylcholine-dependent relaxation in mesenteric arteries

The concentration-response relaxation was determined for Ach and resveratrol. Ach-mediated relaxant responses were significantly diminished in OC rats compared to YC group (p<0.05) with both a decrease in maximal relaxation and a right shift in the concentration response curve (Figure 2). Two-week treatment with resveratrol did not significantly alter relaxation in the young group, however, reservatrol treatment reversed the impaired Ach-mediated relaxant responses in the aged group, such that there were no longer any significent differences with age (Figure 2). Endothelium-independent relaxantation responses using SNP (10-5 M) in YR, OC and OR groups fully relaxed MA and were not significantly different between groups (data not shown).

**Figure 3.**
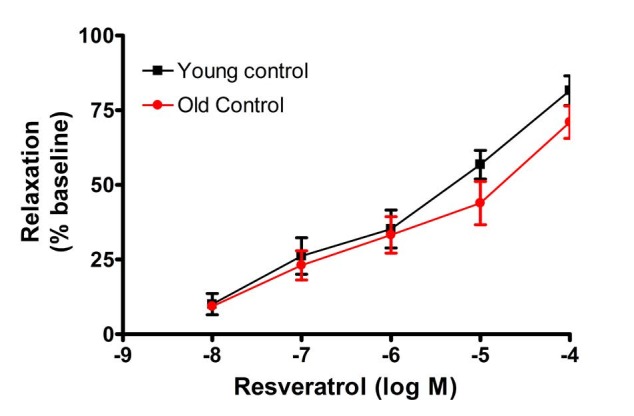
Cumulative dose-response curves to resveratrol-dependent relaxation

In contrast to Ach-mediated relaxation, resveratrol-induced vasodilation was indistinguishable between YC and OC both with respect to sensitivity and effiacy (Figure 3). Again, relaxant responses to SNP (10^-5^ M) were not significantly different between YC and OC groups.

### eNOS and CuZnSOD in the Aorta

Due to insufficient amount of mesenteric arterial tissue, aortas were used for analysis for eNOS and CuZnSOD protein levels. Expression of eNOS in aorta was not significantly different with either age or resveratrol treatment, although there is a trend for an increase with age and with resveratrol (Table 2). Similarly, neither age nor resveratrol had any detectable effect on the expression of CuZnSOD protein (Table 2).

**Table 2.** eNOS and CuZnSOD in the Aorta.

	Young		Old	
	Control	Resveratrol	Control	Resveratrol
eNOS	100 ± 17	130 ± 17	136 ± 19	162 ± 15
CuZnSOD	100 ± 9.1	105 ± 4.6	92.1± 4.7	101 ± 5.9

Data represent the mean ± SE of 8 rats per group. The value of Control for each age is arbitrarily set to 100 with SE adjusted proportionally with remaining groups normalized to the level in respective Control.

### Tyrosine hydroxylase, dopamine beta hydroxylase and NPY in adrenal medul

**Figure 4.**
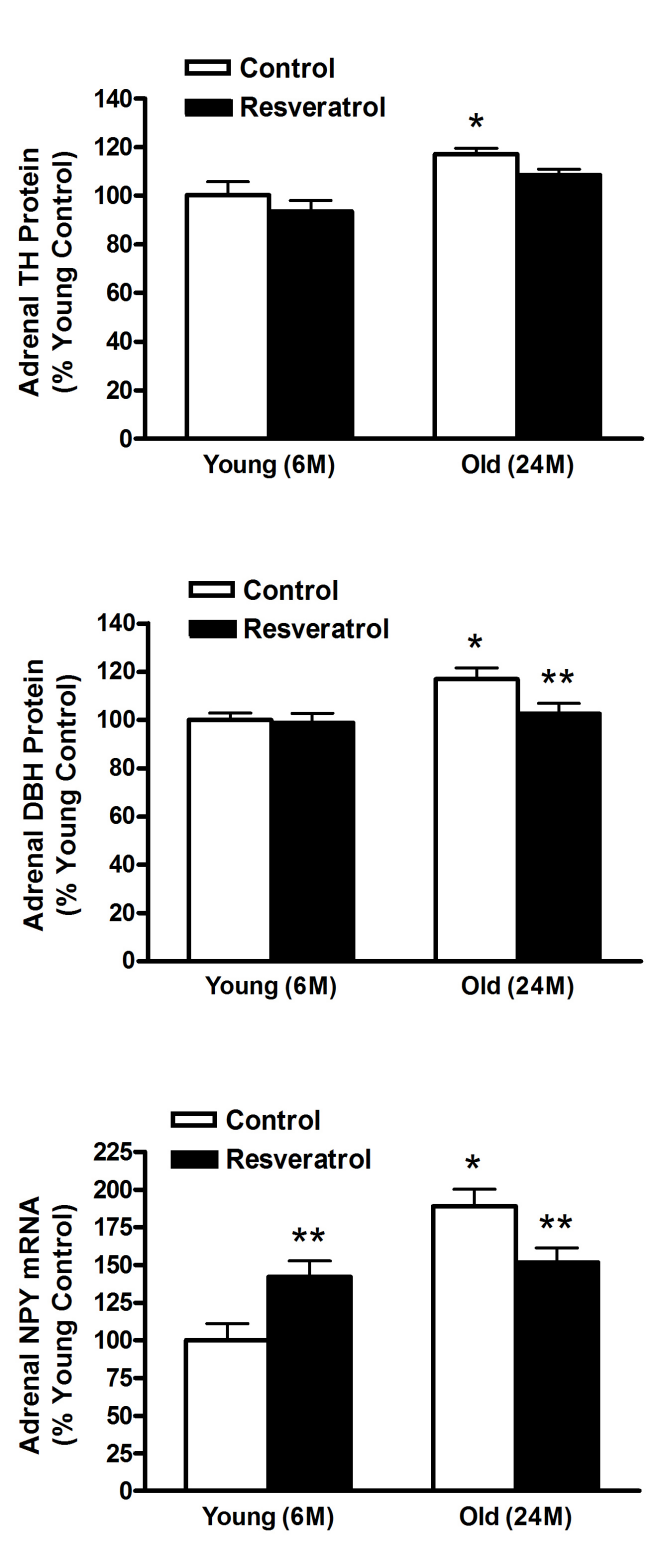
Protein levels of TH (top) and DβH (middle) and mRNA levels of NPY (Bottom) in young and old rats with and without resveratrol treatment.

Tyrosine hydroxylase (TH) and dopamne beta hydroxylase (DβH) protein levels were examined in the adrenal medulla after two weeks of resveratrol treatment. Both TH and DβH protein levels were augmented with age, and for DβH, these elevated levels were restored to young control levels with resveratrol treatment (Figure 4, top and middle, respectively). In addition, mRNA levels of NPY, a peptide co-synthesised and released with cathecolamines from the adrenal medulla, were elevated with age and resveratrol treatment in the old rats decreased NPY expression. Interestingly, resveratrol adminiatration increased NPY expression in the young, such that there was no longer a age related difference between the young and old resveratrol treated rats (Figure 4, bottom).

## DISCUSSION

This study demonstrates that two-week resveratrol treatment reverses impaired endothelium-dependent Ach-mediated relaxant responses in aged rats. In contrast, resveratrol-induced concentration-dependent vasorelaxation was not different between young and aged rats, suggesting an endothelium independent mechanism. Although SOD expression in aorta was not changed either by aging or twoweek resveratrol treatment, eNOS protein levels in aorta tended towards an increase with resveratrol treatment. Finally, our results demonstrated for the first time that resveratrol administration partially normalized adrenal medullary function with age as related to DβH and NPY content.

With respect to vascular activity, the PE-mediated concentration-dependent constriction was right-shifted with age, however, the maximum constriction was similar in young and aged rats. Subsequent Ach-mediated vasodilation demonstrated both a rightward shift in the relaxation-dependent concentration curve and a decrease in maximum relaxation at the Ach concentration of 10-5M. Most interestingly, the short two-week treatment with resveratrol reversed these age-related impairments in Ach-mediated relaxation. In constast, to the diminished vasodilatory responses with age to Ach, resveratrol-mediated concentration-dependent relaxation in both young and old MAs were similar. These data suggest that the resveratrol-mediated relaxation is an endothelium independent mechanism. Previous studies have indicated that resveratrol-induced vasorelaxation may be either endothelium-dependent or endothelium-independent^26,29,30,40^. The endothelium-dependent vasorelaxation is largely attributable to NO, whereas the endothelium-independent relaxation is likely to be mediated by ion channels including voltage-gated K^+^ channels, big Ca^++^-activated K^+^ channels, 4-AP and margatoxin-sensitive K^+^ channels or voltage-gated Ca^++^ channels ^41-43^. Resveratrol might also become incorporated into the smooth muscle membrane, where it could either couple with a membrane receptor^31^ or interact directly with membrane calcium channels^44^, thus inducing endothelium-independent vasorelaxation30. It is well known that polyphenols inhibit cyclic nucleotide phosphodiesterases, which hydrolyse the vasorelaxants cAMP and cGMP45. It is possible that such a mechanism might be involved in the endothelium-independent relaxation induced by resveratrol. Furthermore, the mechanism of resveratrol-induced vasorelaxations may depend on the concentration of resveratrol. Chen and coworkers28 concluded that resveratrol exerts both indirect and direct vasodilatory effects on the blood vessels by NO-mediated and non-NO-mediated mechanisms in rat aortic rings. These authors reported that the former effect is apparent at low resveratrol concentrations (10-30 μM/L) and is blocked by inhibitors of NOS activity, whereas endothelium-independent effects appear at high resveratrol concentrations (>60 μM/L) and are not blocked by endothelial denudation or NOS inhibitors^28^. On the other hand, Naderali and coworkers^30^ found that L-NAME attenuated the responses to resveratrol of arteries from lean animals, but notdietary-obese animals with impaired endothelial function, suggesting resveratrol relaxaton is mediated via NO in lean rodents.

In the present study, resveratol appears to be involved in both endothelium-dependent and endothelium-independent functions. The resveratrol-mediated direct vasorelaxation appears to be an endothelium independent, but resveratol treatment, *in vivo*, improves Ach-mediated endothelium dependent relaxation. Moreover, resveratrol treatment had no effect on the endothelium-independent relaxation induced by SNP. Aged rats^5,6^, similar to dietary-obese rats, have impaired endothelial function, as evidenced by the diminished vasorelaxation to Ach. Resveratrol treatment tended to stimulate the synthesis or availability of NO in MAs, and this may underlie the restoration of Ach-medated relaxation in the aged rats with resveratrol treatment.

With regards to eNOS expression in aging, conflicting data have been reported, showing reduced^9^, unchanged^46^ or increased^11,47^ eNOS protein levels during aging. Therefore, it is possible that changes in eNOS expression and activity are responsible either for preserved NO availability, or for improved vasorelaxation in aged animals with resveratrol treatment. Endothelial cell culture studies demonstrate an upregulation of eNOS expression and activity as a result of acute or short-term resveratrol exposure^48-50^. Animal studies indicated that long-term treatment of resveratrol augmented basal release of NO and nitrite/nitrate levels in aorta, and potentiated endothelial function in healthy animals^37,38^. Another study reported that administration of resveratrol to rabbits with high-cholesterol improved flow-mediated vasodilation and increased plasma NO level^36^. Similar to the present study, Rush and coworkers^34^ reported that resveratrol treatment improved the maximal Ach-induced, endothelium-dependent, NO-mediated relaxation of aorta in spontaneous hypertensive rats^34^. Moreover, this occured in the absence of significant changes in aorta eNOS protein levels^34^. In the present study, the restoration of the Ach-mediated vasorelaxation in aged rats is consistent with resveratrol-induced elevation of eNOS synthesis or NO availability, however, the observed increase in eNOS was minor and without significance. It is possible that there is increased NO availablity or that other mechanisms may be involved.

Possible mechanisms include NO-guanylyl cyclase interactions or subsequent cGMP-dependent mechanism in the MAs smooth muscle cells or changes in oxidative stree or inflamation. Oxidative stress is attributable to excessive production of reactive oxygen species (ROS) and is known to be the key factor in the pathogenesis of age-related vascular dysfunction^5,10^.The inactivation of NO by ROS is recognized to be a crucial factor in reducing NO bioavailability and the development of endothelial dysfunction^51^. Furthermore, resveratrol has been shown to reduce superoxide-mediated NO breakdown, and thus enhance NO bioavailability leading to improved endothelial function^35,38,52^. Although levels of CuZnSOD were not significantly different with age in the present study, these findings do not eliminate the possibility that resveratrol treatment might change the activities of other antioxidant processes.

In addition, inflammation is beleived to play an essential role in the etiology of vascular aging. The pro-inflammatory nuclear transcription factor NF-κB(nuclear factor κB), pro-inflammatory cytokines IL-6 (interleukin-6), TNF-α (tumor necrosis factor-α) and MCP-1 (monocyte chemoattractant protein-1) are increased in older adults^5,7,53^. Resveratrol restored endothelial function in type 2 diabetes by inhibiting TNFα-induced activation of NAD(P)H oxidase and preserving eNOS phosphorilation^35^. In a human coronary arterial endothelial cell study, resveratrol inhibited TNF-α-induced NF-κB activation and inflammatory gene expression^54^. Althoug inflammatory parameters were not examined in the present study, it may speculated that antiinflammatory actions of resveratrol participates in the restroration of age-related impared Ach-mediated vasorelaxation.

The increase in adrenomedullary catecholamine biosynthesis may play an important role in the age-related increase in sympathetic nervous activity and is likely a significant factor in the development of hypertension and cardiovascular diseases with age. Catecholamines are formed from their amino acid precursor tyrosine in the brain, chromaffin cells of the adrenal medulla, and sympathetic nerves. TH catalyzes the hydroxylation of tyrosine, producing dopamine, whereas DβH catalyzes the conversion of catecholamines from the adrenal medulla. We and others have shown that the levels of catecholamine biosynthetic enzymes^15-18,55^ and NPY increase with age in the adrenal medulla^19,20,22,56^. In present study, resveratrol treatment normalized the levels of DβH and NPY in the adrenal medulla of aged rats. Notably, the levels of TH were not changed by resveratrol administration. TH, DβH and NPY are reliable biomarkers of sympathetic nervous system activity, thus resveratrol may partially normalize elevated sympathetic activity with age. Because elevated plasma catecholamine levels may be contributing to the increased prevalence of hypertension in the elderly, this previously unrecognized therapeutic benefit of resveratrol is especially important in the elderly where the higher prevalence of hypertension is associated with elevated morbidity and mortality^1,2^.

## CONCLUSIONS

This study indicates that age-related dysfunction inendothelium-dependent vasodilation in MAs can bereversed with resveratrol treatment. The two-week treatment with resveratrol tends to increase eNOS level in aorta from aged rats, and these changes are associated with improvement of age-associated endothelial function. Direct vasorelaxation by resveratrol is unchanged across age, suggesting that resveratrol-mediated relaxation is endothelial independent. Resveratrol partially reversed the hyperactivity of adrenomedullary function with age by reducing the elevated adrenomedullary DβH and NPY levels. These resveratrol-mediated responses may have a therapeuticpotential in the treatment of cardiovascular diseases and hypertension, especially in the elderly.

## CONFICT OF INTEREST STATEMENT

The authors declare no conflicts of interest.
